# MU-PseUDeep: A deep learning method for prediction of pseudouridine sites

**DOI:** 10.1016/j.csbj.2020.07.010

**Published:** 2020-07-15

**Authors:** Saad M. Khan, Fei He, Duolin Wang, Yongbing Chen, Dong Xu

**Affiliations:** a Informatics Institute, University of Missouri, Columbia, MO 65211, United States; b Department of Electrical Engineering and Computer Science and Christopher S. Bond Life Sciences Center, University of Missouri, Columbia, MO 65211, United States; c School of Information Science and Technology, Northeast Normal University, Changchun 130117, China

**Keywords:** Pseudouridine site prediction, Deep learning, RNA secondary structure

## Abstract

Pseudouridine synthase binds to uridine sites and catalyzes the conversion of uridine to pseudouridine (Ψ). This binding takes place in a specific context and in the conformation of nucleotides. Most machine-learning methods for Ψ site classification use nucleotide frequency as a feature, which may not fully depict the relevant conformation around a Ψ site. Using the power of deep learning and raw sequence, as well as secondary structure features, our tool MU-PseUDeep is designed to capture both the sequence and secondary structure context, which inputs the raw RNA sequence and the predicted secondary structure to two sets of convolutional neural networks. It has shown considerable improvement in Ψ site prediction over existing tools, XG-PseU, PseUI, and iRNA-PseU for both balanced and imbalanced datasets. To the best of our knowledge, this is the most accurate tool for Ψ site prediction. We also used MU-PseUDeep to scan the human transcriptome, which shows that the genes with predicted Ψ sites are enriched in nucleotide and protein binding, as well as in neurodegeneration pathways. The tool is open source, available at https://github.com/smk5g5/MU-PseUDeep.

## Graphical abstract

**Figure:**
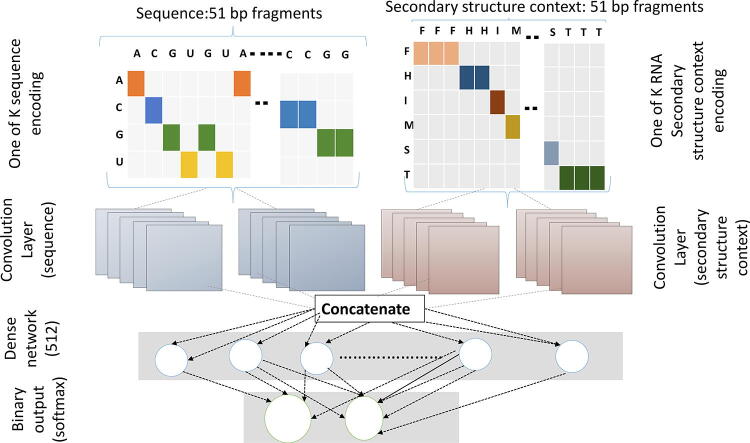

## 1 Introduction

Pseudouridine (Ψ) is one of the most abundant RNA modifications in a cell [1]. Ψ is also known as the fifth nucleotide base of RNA [2]. It results from the isomerization of a uridine base. This process of isomerization is a post-transcriptional mechanism known as pseudouridylation [3], [4], which is catalyzed by pseudouridine synthases (PUS) [5], [6], [7], [8]. Ψ has significant functional and disease implications. For some types of cancers, Ψ provides important biomarkers [9], [10], [11], [12], [13]. The sequencing method (Pseudo-seq) can identify Ψ sites on a large scale at a single nucleotide resolution, but it requires a high sequencing depth as well as multiple biological replicates in order to do so accurately; thus, Pseudo-seq can be very costly [14], [15]. A low-cost alternative is predicting Ψ sites using machine learning. Most machine learning methods developed so far use traditional approaches like support vector machines (SVM). For instance, PPUS, an SVM based method uses nucleotides around Ψ as features [16]. In contrast, iRNA-PseU uses the pseudo-nucleotide composition, including a combination of physicochemical properties of nucleotides and nucleotide densities as features for SVM [17]. Another method, **pse**udo-**u**ridine (Ψ) **i**dentification (PseUI) uses five different kinds of features including nucleotide composition (NC), dinucleotide composition (DC), pseudo dinucleotide composition (pseDNC), position-specific nucleotide composition (PSNP) and position-specific dinucleotide propensity (PSDP), followed by a sequential forward selection strategy to select features for SVM classification of mRNA fragments [18]. While these methods have reasonable performance, there is considerable room for improvement. These tools do not benefit from the latest deep learning techniques, which pose several advantages in comparison to traditional machine learning methods. First, deep learning has been demonstrated to significantly outperform traditional machine-learning methods in multiple domains. Secondly, deep learning reduces the need for feature engineering. Lately, there has been an upsurge in the development of deep learning methods in genomics [19]. Some of these prediction methods have been in the area of RNA modification prediction [20], [21], [22].

**Fig. 1:**
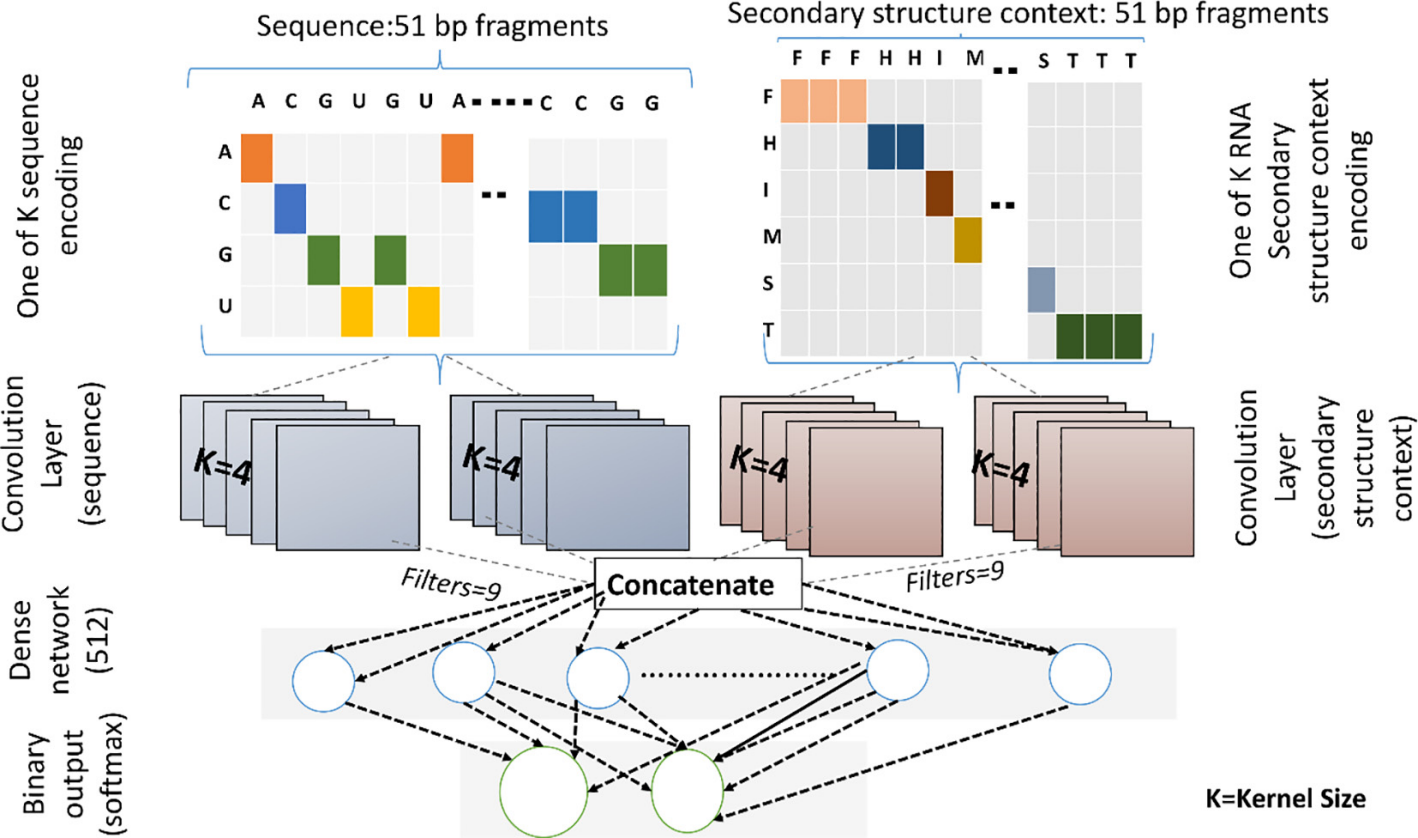
Deep learning architecture for MU-PseUDeep. There are two input layers for sequence and secondary structure. Both layers are one-of-K encoding of a 51-base pair RNA fragment and its secondary structure context. Feature maps for each encoding are generated using two convolutional layers for each of the two encodings. Feature maps are then concatenated and fed into the 512-neuron dense layer. The Final layer is a 2-neuron dense layer with a softmax binary output.

We have developed a deep learning convolutional neural network for the identification of Ψ sites, called MU-PseUDeep. Fig. 1 summarizes the deep learning architecture of MU-PseUDeep used for the classification of Ψ sites. Unlike previous methods employing nucleotide composition and physico-chemical properties, the novelty in this work is to use the secondary structure context of an mRNA fragment as an input feature in addition to the sequence for the input to our deep learning model. Ψ modification plays an important role in stabilizing the secondary structure of RNA. Ribonucleoproteins depend strongly on the structural context of RNA when they catalyze the isomerization of uridine to Ψ [23]. Thus, it is reasonable to hypothesize that the secondary structure is crucial for the identification of Ψ sites. To the best of our knowledge, secondary structure context has never been used for this problem, although deep learning approaches do exist, which utilize a secondary structure context to predict RNA-protein sequence and structure binding [18]. By including secondary structure features, we significantly improved the prediction of Ψ sites in comparison to other available methods. Very recently, a new study explored a deep learning approach to predict Ψ sites called iPseU-CNN [24]. Since no source code or webserver was available for this approach, a direct comparison is impossible; however, we have compared our method with a sequence-only CNN, the architecture of which closely resembles that of iPseU-CNN. Compared to the sequence-only CNN which only uses RNA sequence fragment encoded with One-of-K encoding, our method which combines both sequence and secondary structure information shows significant improvements. We have made predictions using the MU-PseUDeep model for human, mouse and yeast datasets. We have also identified potential Ψ sites by conducting a transcriptome-wide prediction of a human transcriptome at >0.99 precision threshold, to explore the functional importance of Ψ in mRNA. These predicted Ψ sites may provide useful hypotheses for experimental validations.

## 2 Materials and methods

### 2.1 Data collection and pre-processing

The Ψ site information was downloaded from RMBase v2.0 [23] for all three species, namely human, mouse, and yeast. For each of the three species, we extracted the Ψ and surrounding 25 bases upstream and downstream nucleotides using BEDTools [24] with reference files of three species, hg19 (human), mm10 (mouse), and sacCer3 (yeast). To create the negative dataset, we collected those regions of RNA that did not contain any experimentally validated Ψ sites. Since in nature, Ψ sites are relatively rare, the number of negative samples in our data is 10 times larger than the number of positive samples, which is a classical imbalance machine learning problem. We did a 10-fold stratified split of positive and negative RNA samples into an 80:20 ratio for training and testing data to maintain the same class ratio in training and testing sets using pandas and Scikit-learn [25]. We reduced the sequence identity between training and testing sets for each fold using cd-hit-est-2d with a minimum sequence identity threshold (0.8) (allowed by cd-hit for RNA sequence with a word length of 4) [26].To further reduce the sequence identity, we globally aligned the remaining training set against the test set using the Needleman-Wunsch algorithm and removed the sequences from the training set that had >60% sequence identity with the test set. The high sequence identity was further removed within the test set using cd-hit-est at the above-defined sequence identity threshold and word size.

For the processed sequence data, the abstract secondary structure dot-bracket notation was generated using the *RNAshapes* package [27], [28]. The dot-bracket notation was further converted into secondary structure context using EDeN (https://github.com/fabriziocosta/EDeN), a neighborhood subgraph pairwise distance kernel-based method for explicit feature representation of graphs. The RNA secondary structure context is represented by six generic sub-shapes, namely Stem (S), multi-loops (M), hairpins (H), internal loop (I), dangling start (F), and dangling end (T). Each 51 bp RNA fragment was coded into a secondary structure context, where each nucleotide was coded into one of the above-mentioned sub shapes. Sequence data was converted into a one-of-K encoding binary matrix of size 51 × 4, where 51 is the length of the fragment of 4 nucleotides. Similarly, the secondary structure was encoded into a one-of-K encoding binary matrix of size 51 × 6, where 51 is the length of the fragment with six sub-shapes of the RNA fragment.

### 2.2 Deep learning architecture of MU-PseUDeep

For each input (sequence and secondary structure), a pair of 1D CNN was used. The first layer of sequence input (seq_1) and secondary structure input (sec_1) both have a filter size of 5 and a kernel size of 10. Similarly, the second 1D CNN layer for both sequence input (seq_2) and secondary structure input (sec_2) has a filter size of 9 and a kernel size of 4. The kernel initializer for each 1D CNN layer was ‘glorot_normal.’ The kernel regularizer weight for each layer (rounded to four decimal places) followed 0.0321 (seq_1), 0.01608 (seq_2), 0.00109 (sec_1) and 0.0340 (sec_2). Dropout rate for each layer was as follows: 66.5% (seq_1), 3.8% (seq_2), 74.5% (sec_1) and 36.9% (sec_2). All layers had a ‘PRelu’ activation function. All layers were concatenated and fed into the dense layer with a ‘softplus’ activation function. A stochastic gradient was used as the optimization algorithm with a learning rate of 0.0137. A binary cross-entropy was used as a loss function with an early-stop patience of 20 and a model checkpoint serving as a callback for fitting the model. The batch size was 32 and the number of epochs was set to 500. The total number of trainable parameters in the network was 661,118. The model was implemented in Keras version 2.2.2 with a Tensorflow (1.10.1) backend [29].

### 2.3 Hyperparameter optimization

A hyperparameter optimization of various hyperparameters was carried out using Hyperas (https://github.com/maxpumperla/hyperas), a convenience wrapper for Hyperopt (https://github.com/hyperopt/hyperopt), and a distributed asynchronous hyperparameter optimization library. A tree-structured Parzen estimator approach was used to optimize the models by maximizing each model’s F1-score on validation data for a single fold [30]. We optimized several hyperparameters of our deep learning architecture including “dropout-rate,” “kernel regularizer weight,” “optimization algorithm,” and “learning rate for the optimizer.” The performance of the top 10 hyperparameter-optimized models on our test data is shown in Supplementary Fig. S1.

### 2.4 Bootstrapping

A bootstrapping approach was applied, like the one used by Wang et al. (2017). In this case, we divided our negative samples into *N* bins. Each bin was the same size as the number of samples in the positive class and was iterated when training the model with the positive class. The final results were calculated by averaging the results from each iteration of every fold [31], [32], [33].

### 2.5 Transfer learning

A bootstrapped hyperparameter optimized human model was further finetuned for transfer learning on yeast and mouse data. All layers were kept fixed/untrainable except for each of the two 1D CNN layers and dense 512 neuron layers. The learning rate of the stochastic gradient descent algorithm was reduced from 0.0137 to 0.0086.

### 2.6 Human transcriptome scanning

The human transcriptome was obtained using BedTools from the hg19 genome and gencode gtf file. The coding sequences were converted from DNA to RNA based on their strand. The positive pseudouridine sites from RMBase were masked with BedTools along with the 25 bases flanking upstream and downstream. Running windows of 51 base pair fragments were generated using SeqKit [34]. Only those fragments with uridine at their center were considered for further prediction. A precision threshold of >0.99 was used to predict whether the uridine site is a potential Ψ site. The GO, pathway, and disease enrichment was performed for genes containing the predicted sites using clusterProfiler [35]. Network construction was based on GO semantic similarity with each edge representing the semantic similarity score between two genes. The GO semantic similarity scores were calculated using GOSemSim [36], and the network construction was done using Cytoscape [37] and a ClueGO plugin [38]. Motif visualization was based on ggseqlogo [39].

## 3 Results

**Fig. 2:**
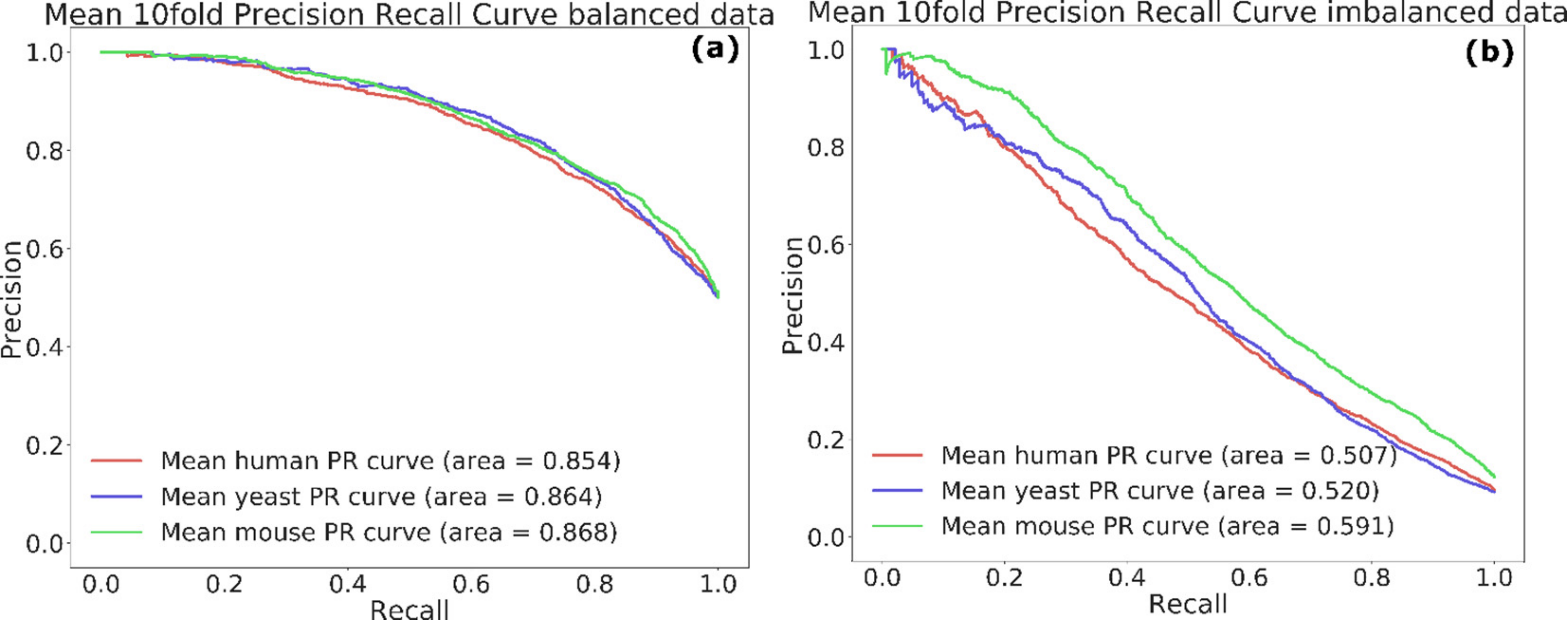
Precision recall Curves. Mean Precision recall curves over 10 folds for (a) balanced and (b) imbalanced data for human, yeast and mouse, respectively.

We compared MU-PseUDeep, which used both sequence and secondary structure context as features, with the one using the sequence-only context (a deep learning model which closely resembles iPseU-CNN) or only the secondary structure context as input. The results of MU-PseUDeep indicate a significant improvement in performance in comparison to either only-sequence CNN or only secondary structure CNN. The improvement was by 3–4% for accuracy and F1 and up to 9% for sensitivity in the balanced dataset in comparison to sequence CNN (which had proved to be better than a secondary CNN structure). Similarly, for the imbalanced dataset, our combined model outperformed the sequence CNN with a 2% accuracy. The improvement was also 2% for F1, up to 4% for MCC, and up to almost 7% for sensitivity as shown in Table S1. Fig. 2 shows the Precision-recall curves of the optimized models for all three species for both balanced and imbalanced test data.

### 3.1 Comparing MU-PseUDeep with other methods

**Fig. 3:**
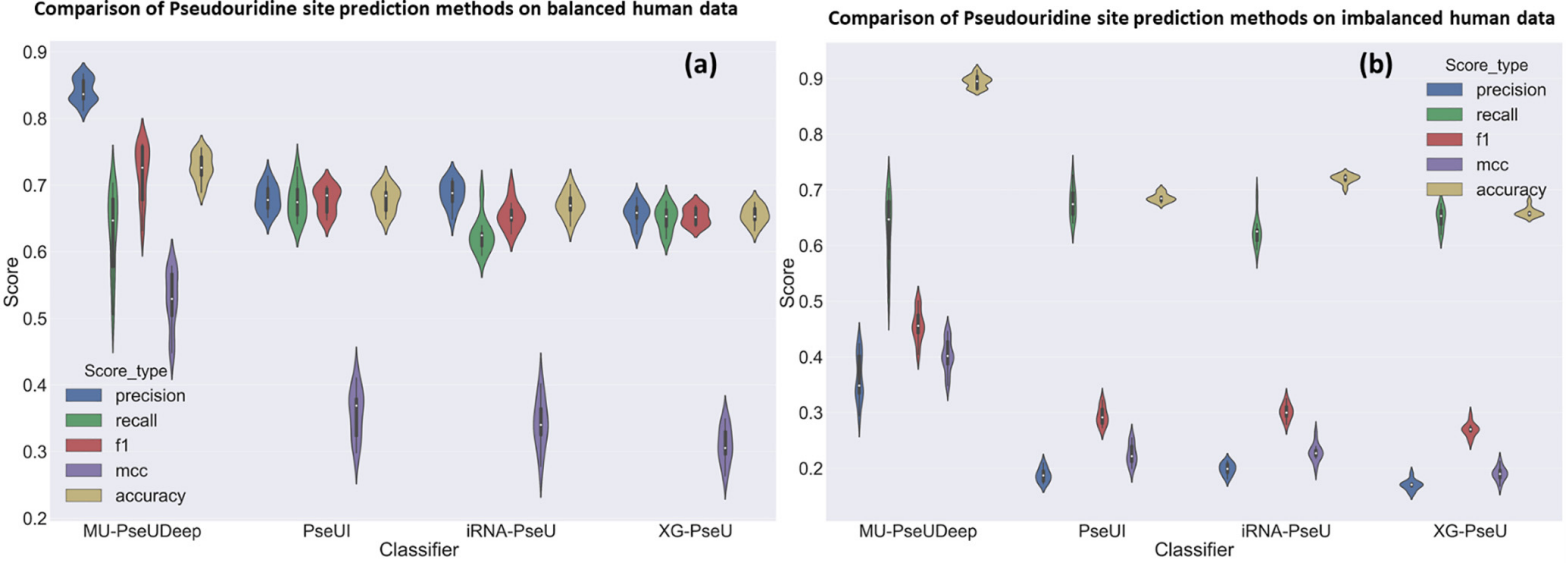
Performance Comparison. This deep learning model was compared with three available methods for Ψ site classification over various performance metrics. The deep learning model performance shows significant improvements over other methods with respect to the various performance metrics for both (a) balanced data and (b) imbalanced data.

**Fig. 4:**
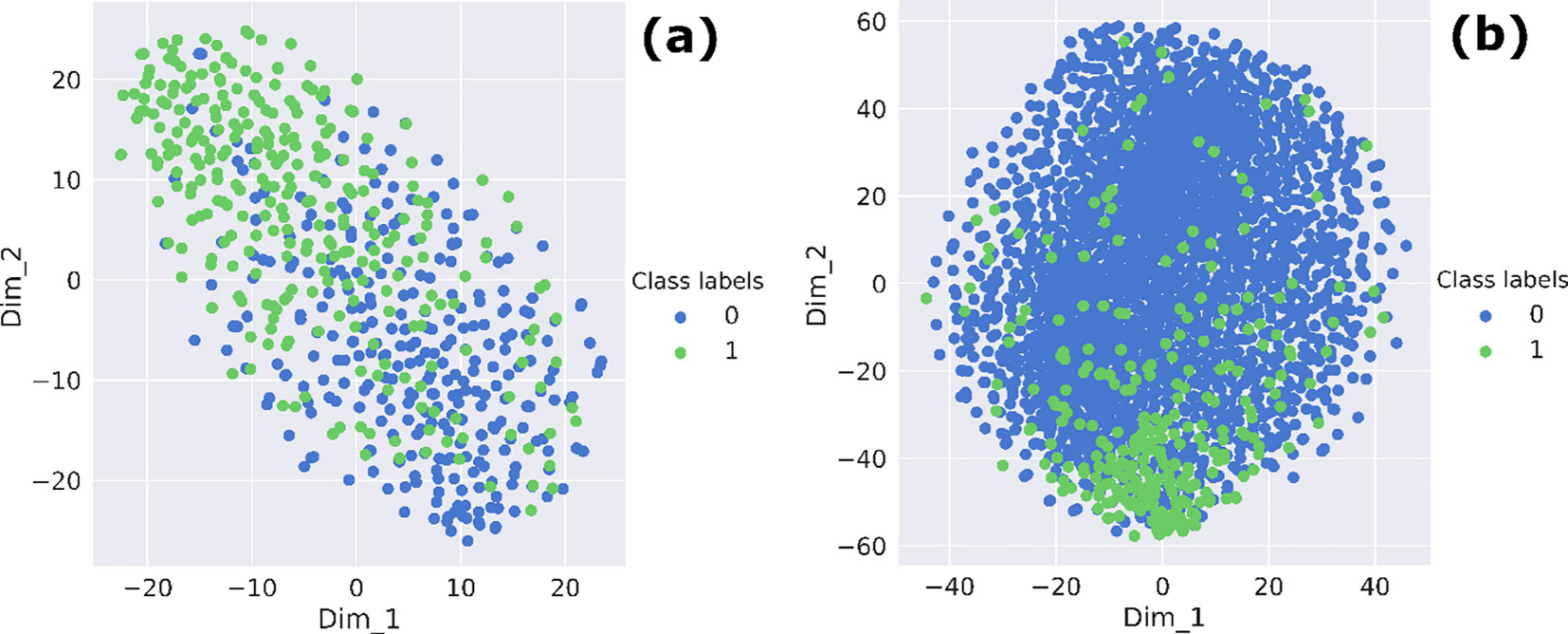
t-SNE plots. Feature map of the last layer of deep learning network. (a) shows the t-SNE plot, which represents the model’s classification efficacy in separating the positive (green) and negative (blue) classes on the balanced test data and (b) the t-SNE plot, which represents the last feature map of the deep learning network on the imbalanced test data. (For interpretation of the references to colour in this figure legend, the reader is referred to the web version of this article.)

**Fig. 5:**
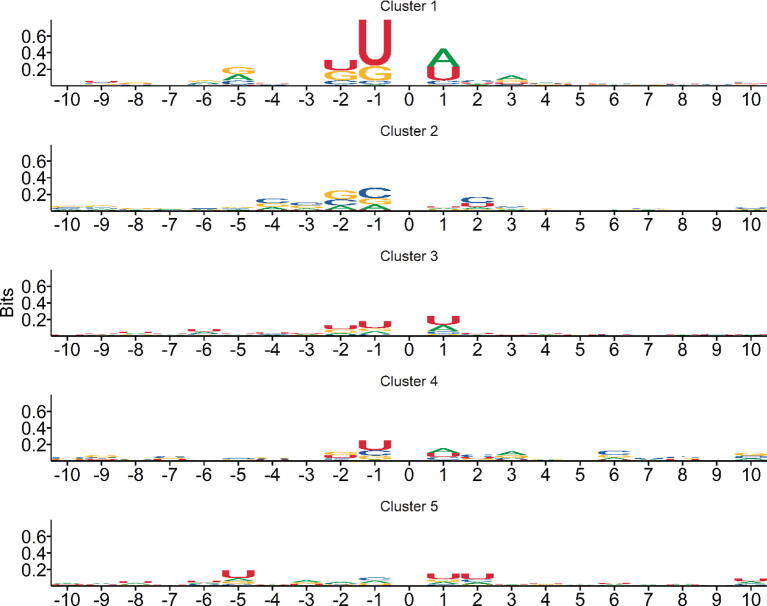
Positive class sequence logos. Positive class sequences were clustered based on the output of the last feature map of deep learning model into 5 clusters using k-means clustering. Only the 10 bases upstream and downstream of Ψ are shown where index 0 is the Ψ site. In the above sequence logo figure, the y-axis represents the entropy or Bits which represent the total information content for a particular position depending on the size of the logos.

Our MU-PseUDeep method with both sequence and secondary structure context features was further compared with the published Ψ site prediction methods namely PseUI (He et al., 2018), iRNA-PseU (Chen et al., 2016) and XG-PseU (Liu et. al., 2019) for human, mouse, and yeast datasets, respectively. For each species, the average 10-fold comparison results are shown in Table 1 for both balanced and imbalanced datasets. For balanced human data, the performance metrics of our model in comparison to PseUI [18] improved on average by 7% for accuracy, 10% for F1 score, 46% for MCC, 5% for sensitivity, and 19% for specificity. Similarly, in comparison to iRNA-PseU [17], the performance metrics of our model improved by 8% for accuracy, 14% for F1, 53% for MCC, and 13% for sensitivity and specificity. Likewise for XG-PseU [40], the accuracy improved by 11%, F1 score by 14%, MCC by 69.5%, sensitivity by 9.2%, and specificity by 22.9%. For the imbalanced data, the performance metrics improved in comparison to PseUI by 30% for accuracy, 41% for F1, 64% for MCC, 5% for sensitivity, and 18% for specificity. In comparison to iRNA-PseU, our method improved by 24% for accuracy, 37% for F1, 61% for MCC, 13% for sensitivity, and 11% for specificity as shown in Table 1 and Fig. 3. Correspondingly, in comparison to XG-PSeU, we saw improvements in accuracy by 35.6%, F1 score by 54.2%, MCC score by around 94.2%, and specificity by 23.4%. Similar improvements were noticed for both the mouse and yeast data as well, as shown in Table 1 and Figures S2–S6. The performance of the MU-PseUDeep model was further assessed by visualizing t-SNE plots of the feature map of the deep learning model. Fig. 4(a) and (b) shows a good separation between positive and negative classes. Similar results were observed for mouse and yeast datasets as shown in Figs. S7 and S8. We clustered the last feature map of our deep learning model on the whole positive dataset as shown in Fig. S9. Subtle differences can be seen between clusters of fragments for nucleotides surrounding the Ψ site within the positive class as shown in Fig. 5, and Figs. S10 and S11 for human, mouse and yeast, respectively. Furthermore, using secondary structure context, we improved the sensitivity of our model.

**Table 1:** Prediction performance of MU-PseUDeep against other available methods.

Data type	Species	Method	Accuracy	F1	MCC	Sensitivity	Specificity
Balanced	Human	**MU-PseUDeep**	**0.726 ± 0.0203**	**0.745 ± 0.041**	**0.524 ± 0.043**	**0.709 ± 0.061**	**0.810 ± 0.0203**
		PSEUI	0.678 ± 0.017	0.677 ± 0.018	0.357 ± 0.034	0.675 ± 0.026	0.681 ± 0.0250
		iRNA-PseU	0.670 ± 0.017	0.654 ± 0.019	0.341 ± 0.034	0.625 ± 0.024	0.715 ± 0.0205
		XG-PseU	0.654 ± 0.013	0.653 ± 0.012	0.309 ± 0.027	0.649 ± 0.019	0.659 ± 0.028
Imbalanced		**MU-PseUDeep**	**0.894 ± 0.0108**	**0.415 ± 0.028**	**0.369 ± 0.029**	**0.709 ± 0.0617**	**0.815 ± 0.0275**
		PSEUI	0.685 ± 0.007	0.293 ± 0.015	0.225 ± 0.018	0.675 ± 0.0263	0.686 ± 0.0076
		iRNA-PseU	0.720 ± 0.0074	0.301 ± 0.0131	0.228 ± 0.0154	0.625 ± 0.0250	0.730 ± 0.0087
		XG-PseU	0.659 ± 0.008	0.269 ± 0.012	0.190 ± 0.012	0.649 ± 0.019	0.660 ± 0.009
Balanced	Mouse	**MU-PseUDeep**	**0.760 ± 0.0306**	**0.771 ± 0.0338**	**0.537 ± 0.0524**	**0.800 ± 0.0791**	**0.730 ± 0.0826**
		PSEUI	0.737 ± 0.0135	0.748 ± 0.0102	0.477 ± 0.0259	0.779 ± 0.0114	0.696 ± 0.0291
		iRNA-PseU	0.713 ± 0.0208	0.733 ± 0.0160	0.432 ± 0.0398	0.788 ± 0.0152	0.638 ± 0.0404
		XG-PseU	0.726 ± 0.009	0.742 ± 0.008	0.456 ± 0.018	0.788 ± 0.013	0.664 ± 0.021
Imbalanced		**MU-PseUDeep**	**0.854 ± 0.0191**	**0.4355 ± 0.0355**	**0.378 ± 0.0307**	**0.800 ± 0.0791**	**0.734 ± 0.0651**
		PSEUI	0.704 ± 0.0060	0.3914 ± 0.0155	0.3218 ± 0.0135	0.779 ± 0.0113	0.6934 ± 0.006
		iRNA-PseU	0.662 ± 0.0078	0.363 ± 0.0154	0.288 ± 0.0152	0.788 ± 0.0159	0.644 ± 0.007
		XG-PseU	0.683 ± 0.007	0.377 ± 0.017	0.306 ± 0.014	0.788 ± 0.013	0.668 ± 0.007
Balanced	Yeast	**MU-PseUDeep**	**0.768 ± 0.0256**	**0.762 ± 0.0296**	**0.546 ± 0.036**	0.742 ± 0.0667	**0.798 ± 0.0560**
		PSEUI	0.716 ± 0.0192	0.732 ± 0.0167	0.436 ± 0.0378	0.777 ± 0.0234	0.655 ± 0.0355
		iRNA-PseU	0.742 ± 0.0202	0.750 ± 0.0178	0.485 ± 0.04007	0.775 ± 0.0137	0.708 ± 0.0295
		XG-PseU	0.749 ± 0.0206	0.755 ± 0.0194	0.499 ± 0.0412	0.773 ± 0.0262	0.724 ± 0.0355
Imbalanced		**MU-PseUDeep**	**0.869 ± 0.0193**	**0.397 ± 0.0389**	**0.360 ± 0.0302**	0.742 ± 0.0667	**0.788 ± 0.0525**
		PSEUI	0.665 ± 0.0080	0.299 ± 0.0175	0.255 ± 0.0149	0.776 ± 0.0235	0.654 ± 0.0104
		iRNA-PseU	0.707 ± 0.0099	0.327 ± 0.0193	0.289 ± 0.0162	0.774 ± 0.0129	0.700 ± 0.0104
		XG-PseU	0.714 ± 0.0106	0.332 ± 0.0208	0.294 ± 0.0219	0.773 ± 0.0262	0.708 ± 0.0103

### 3.2 Identification of Ψ sites in human transcriptome

**Fig. 6:**
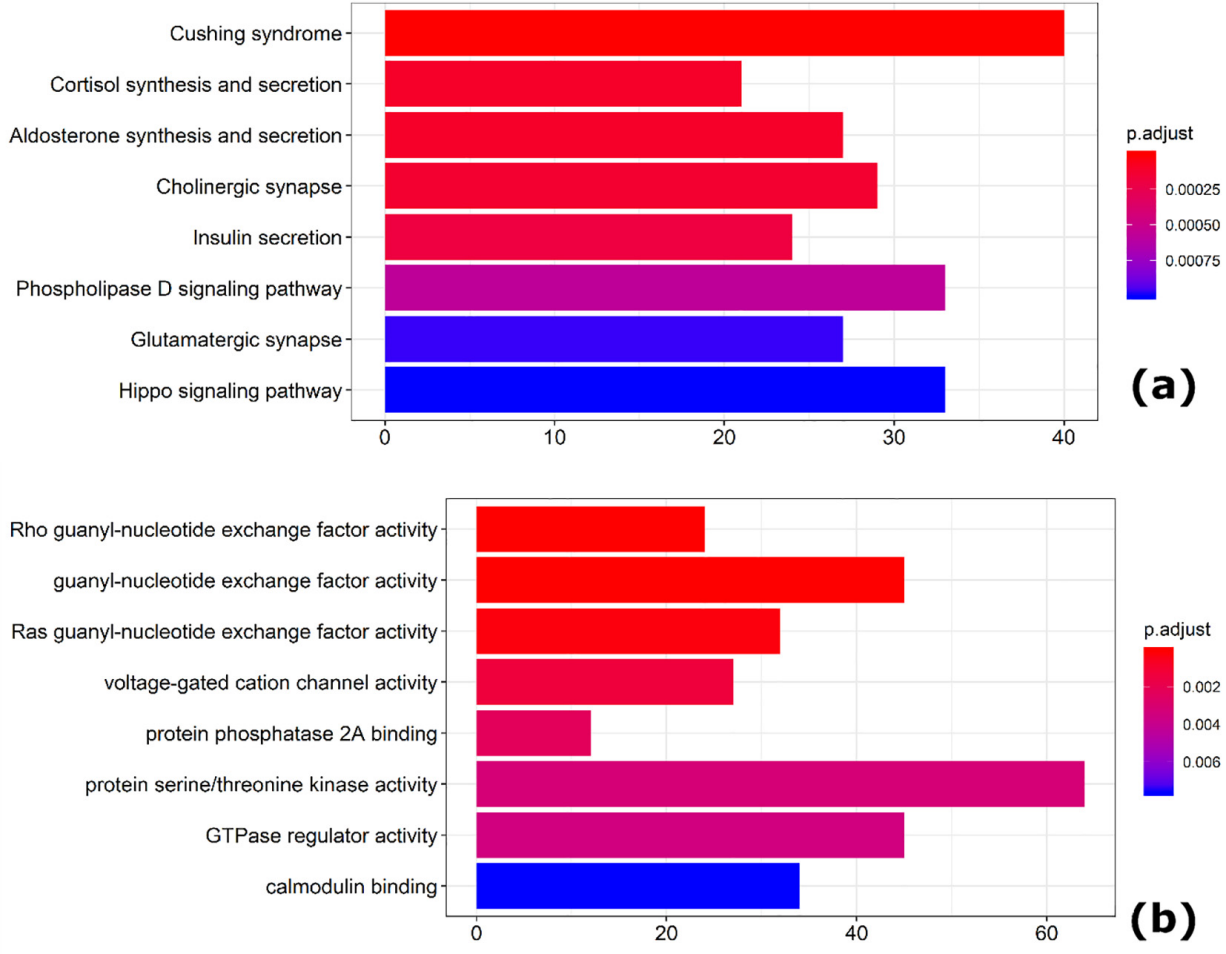
Pathway enrichment of predicted Ψ site containing genes. After transcriptome scan the genes containing one or more Ψ sites were examined for gene enrichment in pathways, GO ontology and disease enrichment (Fig. S12) (a) the KEGG pathway enrichment of genes containing one or more predicted Ψ sites at >0.99 prediction threshold and (b) gene ontology (GO) molecular function enrichment of genes containing one or more predicted Ψ sites.

We applied MU-PseUDeep to scan the protein-coding genes in the human transcriptome, which identified 2441 genes with one or more predicted Ψ site at the >0.99 precision threshold (details about genomic region can be found in the supplemental Excel file, “Supplemental_table2.csv”). Among them, 284 genes already had one or more known Ψ site as documented in the RMBase. Functional enrichment of all 2441 genes indicated a few interesting categories, namely ‘guanyl-nucleotide exchange factor activity,’ ‘DNA-binding,’ ‘Protein-binding,’ as shown in Fig. 6. Likewise, the KEGG pathway enrichment against the whole gene-set background resulted in several enriched pathways namely ‘Cushing syndrome,’ ‘cortisol synthesis,’ and ‘Hippo signaling pathway.’ Network visualization of some of the most functionally similar genes based on GO semantic similarity score >0.9 indicates how some of the genes which contain known as well as predicted Ψ sites are strongly connected with the ones which have one or more predicted Ψ site. Some of these genes belong to a specific functional/pathway category as shown in Fig. 5, and Supplementary Figs. S12 and S13**.** Some of these genes are enriched in signaling pathways that have potentially an important role to play in brain functions. Our prediction results justify our hypothesis of the potential importance of RNA secondary structure that is critical for PUS (pseudouridine synthase) to successfully catalyze the Pseudouridylation reaction.

## 4 Discussion and conclusion

Our transcriptome scanning results indicate how most genes enriched for predicted Ψ sites have a role in nucleotide and protein binding. In addition, enrichment of these genes in certain cancer pathways, cholinergic pathways, and calcium and potassium gated ion channel activity implies their potential involvement in some types of cancers as well as brain disorders, which has already been demonstrated for Ψ in t-RNAs and r-RNAs.

Literature mining for Ψ along with any of the PubMed search terms related to enriched pathways, diseases, and molecular functions, as well as biological components and cellular compartments revealed some interesting relationships between Ψ and insulin secretion [41]. One of the enriched molecular function terms is the “guanyl-nucleotide exchange factor” activity. It is known that Ψ and other modified ribonucleosides play an important role in inter-nucleotide bond formation by means of guanyl-specific ribonucleases [42]. Ψ is also known to bind protein phosphatase in some bacterial species [43], [44], [45]. Previous research has also indicated the importance of Ψ in regulating neuronal functions [46], which is corroborated by the enrichment of biological processes shown in Fig. S12(a) and (b). Disease gene network enrichment articles have shown a relationship of Ψ to brain disorders as shown in Fig. S12(c), which is consistent with an earlier study suggesting that Ψ has a role in mental retardation [12]. Other evidence of Ψ’s role in neural disorders is by elevated levels of Ψ in the urine of mild to moderate-severe Alzheimer patients [47]. Pseudouridylation has also been linked to high oxidative stress, which is known to be one of the risk factors for increased neurodegeneration [48]. Ψ modification has also been linked to myotonic dystrophy [49], a type of genetic neuromuscular disease, which is associated with intellectual disability—another enriched term from the disease gene network database for our list of genes containing putative Ψ sites.

This is perhaps the only method that utilizes RNA secondary structure context along with sequence as featured in Ψ site prediction using a deep learning architecture. We significantly improved upon the performance of existing methods by incorporating both secondary structure and sequence information. Our method has shown considerable improvement in terms of accuracy, F1 score, MCC, sensitivity, and specificity for both balanced and imbalanced datasets over the existing tools including PseUI and iRNA-PseU.

## Declaration of Competing Interest

The authors declare that they have no known competing financial interests or personal relationships that could have appeared to influence the work reported in this paper.
