# Supplementary material for: MU-PseUDeep: A deep learning method for prediction of pseudouridine sites
Source: Comput Struct Biotechnol J. 2020 Jul 15;18:1877–83. doi: 10.1016/j.csbj.2020.07.010 (PMC7387732; doi:10.1016/j.csbj.2020.07.010)
Supplement: Supplementary data 1 [file mmc1.docx]

MU-PseUDeep: a Deep Learning Method for Prediction of Pseudouridine Sites

Saad M. Khan^a^, Fei He^b,c^, Duolin Wang^b^, Yongbing Chen^c^ and Dong Xu^a,b*^

^a^Informatics Institute, University of Missouri, Columbia, MO 65211, USA

^b^Department of Electrical Engineering and Computer Science and Christopher S. Bond Life Sciences Center, University of Missouri, Columbia, MO 65211, USA

^c^School of Information Science and Technology, Northeast Normal University, Changchun, 130117, China

*To whom correspondence should be addressed.

Table S1. Comparison of different network structures including only sequence CNN vs Seq + sec CNN

| Data type | CNN type | Accuracy | F1 | MCC | Sensitivity | Specificity |
| --- | --- | --- | --- | --- | --- | --- |
| Balanced | Secondary structure  CNN | 0.527 | 0.672 | 0.106 | 0.656 | 0.450 |
|  | Sequence only CNN | 0.700 | 0.716 | 0.497 | 0.649 | 0.837 |
|  | Seq + Sec CNN | 0.726 | 0.745 | 0.524 | 0.709 | 0.810 |
| Imbalanced | Secondary structure  CNN | 0.853 | 0.186 | 0.05 | 0.790 | 0.267 |
|  | Sequence only CNN | 0.88 | 0.412 | 0.357 | 0.649 | 0.838 |
|  | Seq + Sec  CNN | 0.894 | 0.415 | 0.369 | 0.709 | 0.815 |

**
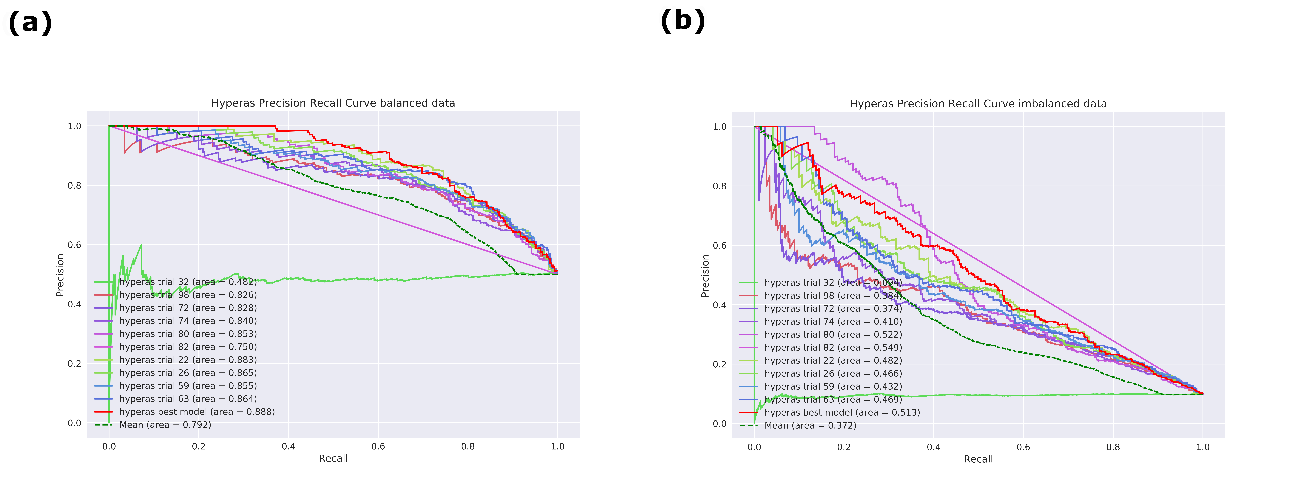
**

**Fig. S1. Hyper-parameter optimization.**  Performance of top ten Hyperas models on **(a)** balanced data and **(b)** imbalanced data.

**
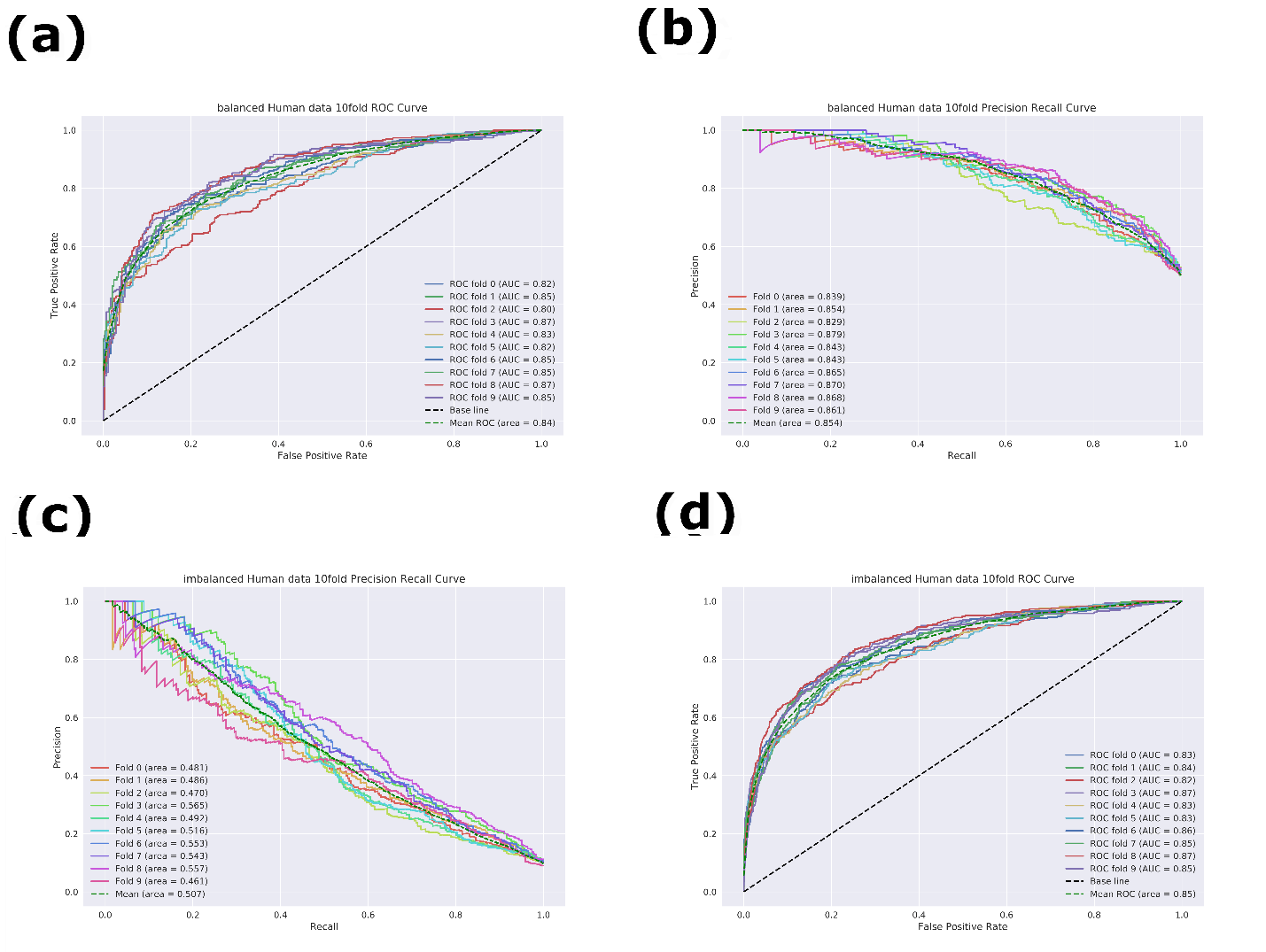
**

**Fig. S2. ROC (a) and (d) and Precision-recall (b) and (c) curves for 10-fold human balanced (a) and (b) and imbalanced data sets (c) and (d).**

**
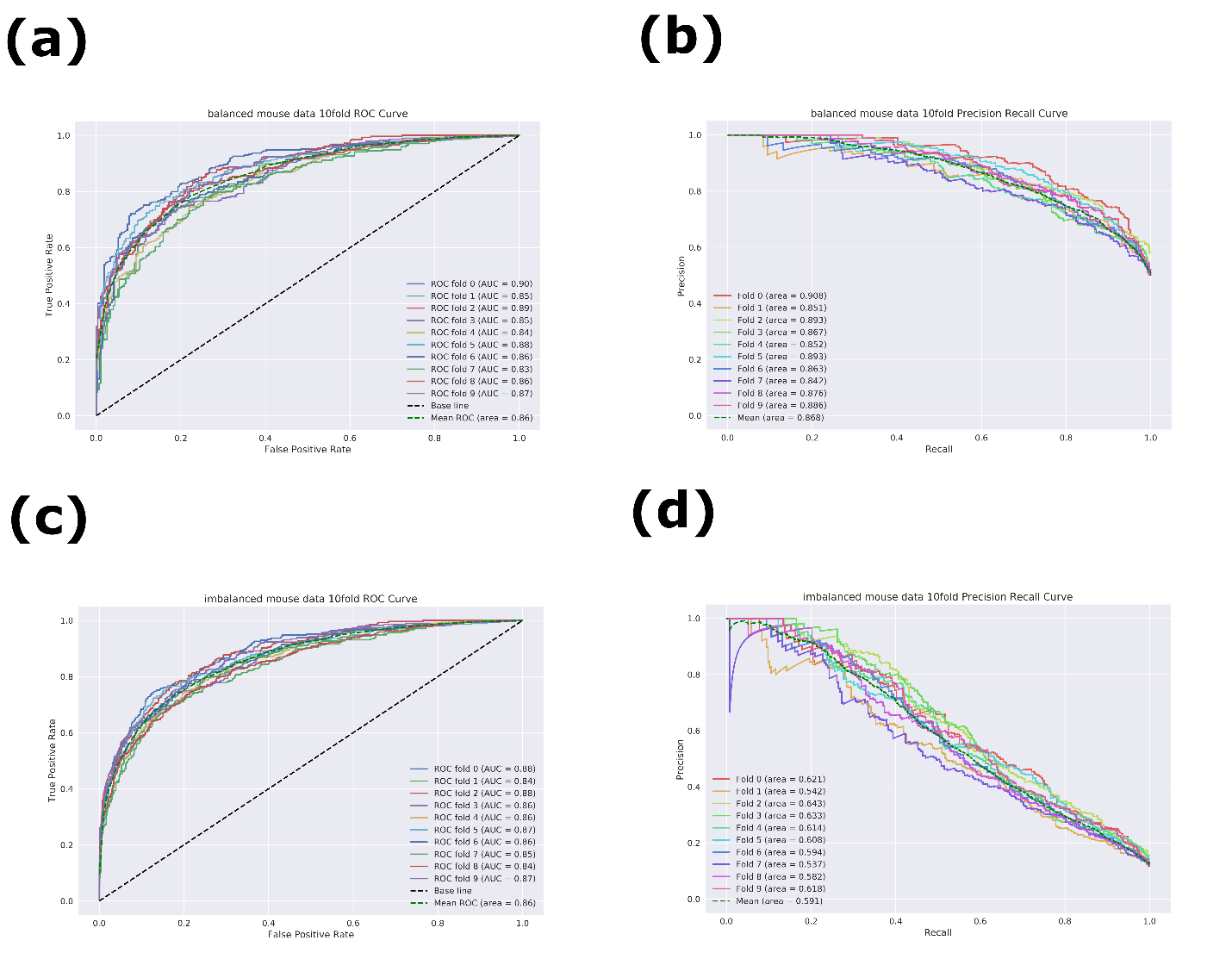
**

**Fig. S3. ROC (a) and (c) and Precision-recall (b) and (d) curves for 10-fold mouse balanced (a) and (b) and imbalanced data sets (c) and (d).**

**
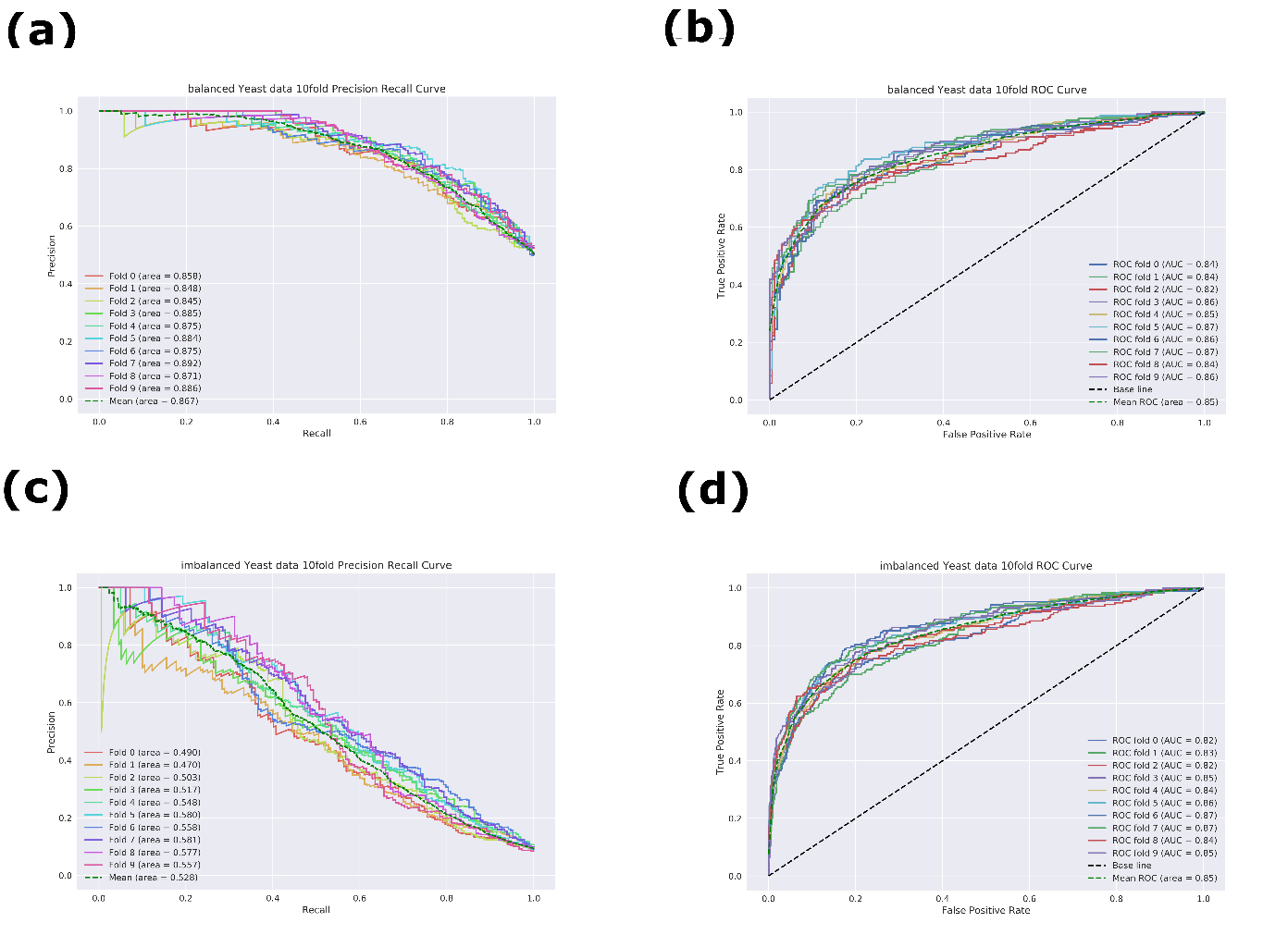
**

**Fig. S4. ROC (b) and (d) and Precision-recall curves (a) and (c) for 10-fold yeast balanced (a) and (b) and imbalanced data sets (c) and (d).**


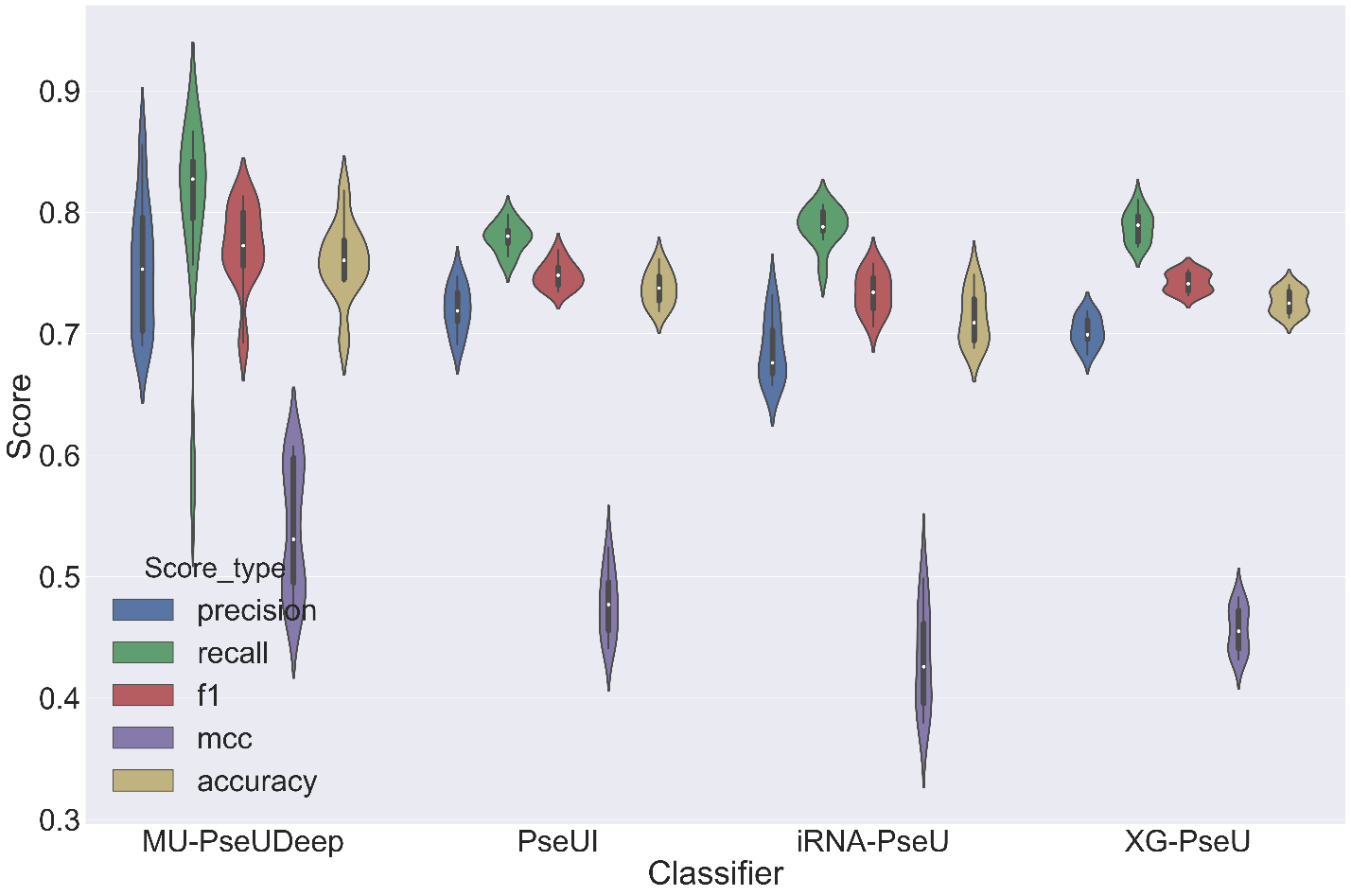


**(a)**

**
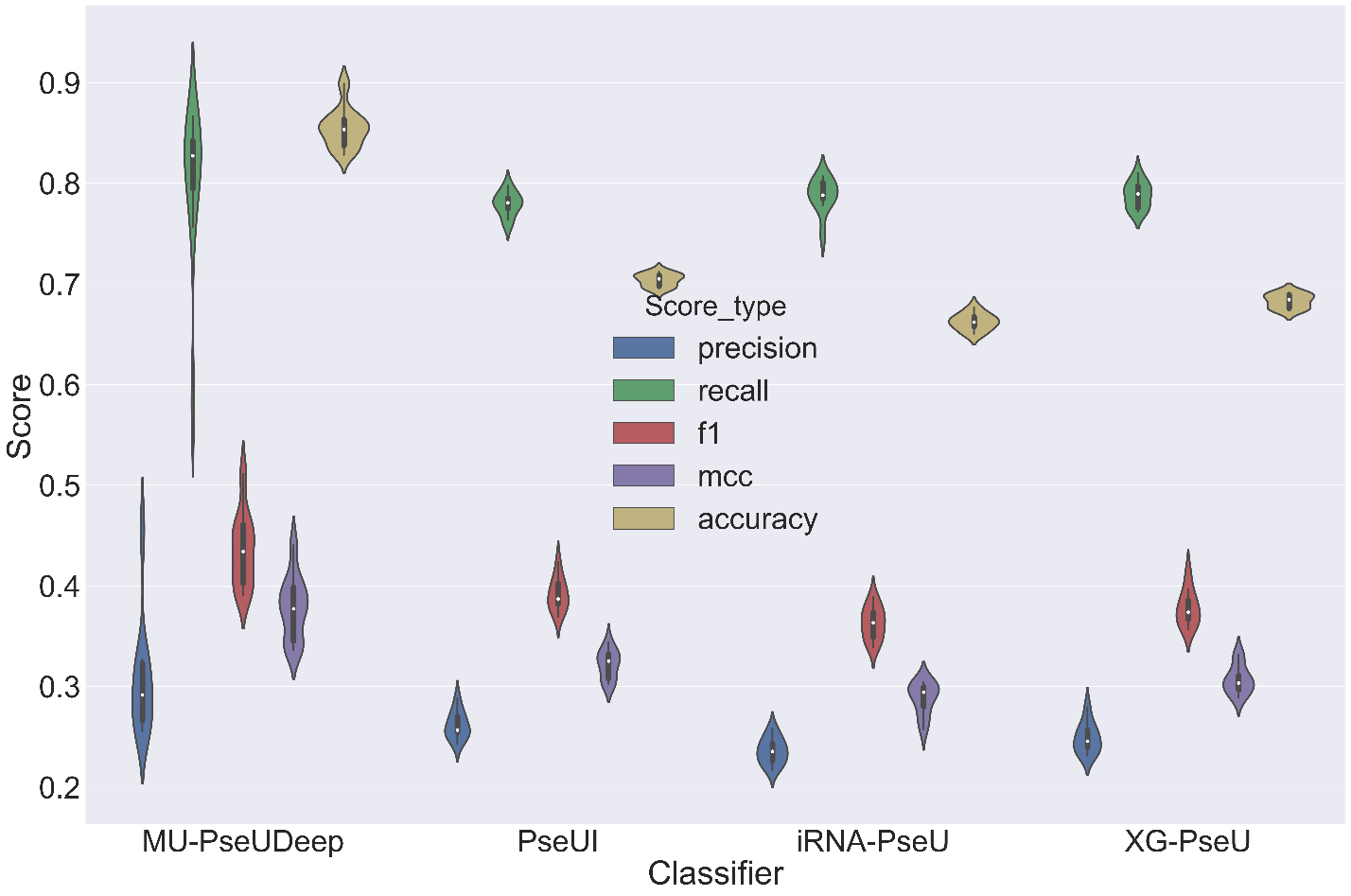
**

**(b)**

**Fig. S5. Performance comparison of deep learning method with other published methods on (a) balanced mouse data and (b) imbalanced mouse data.**

**
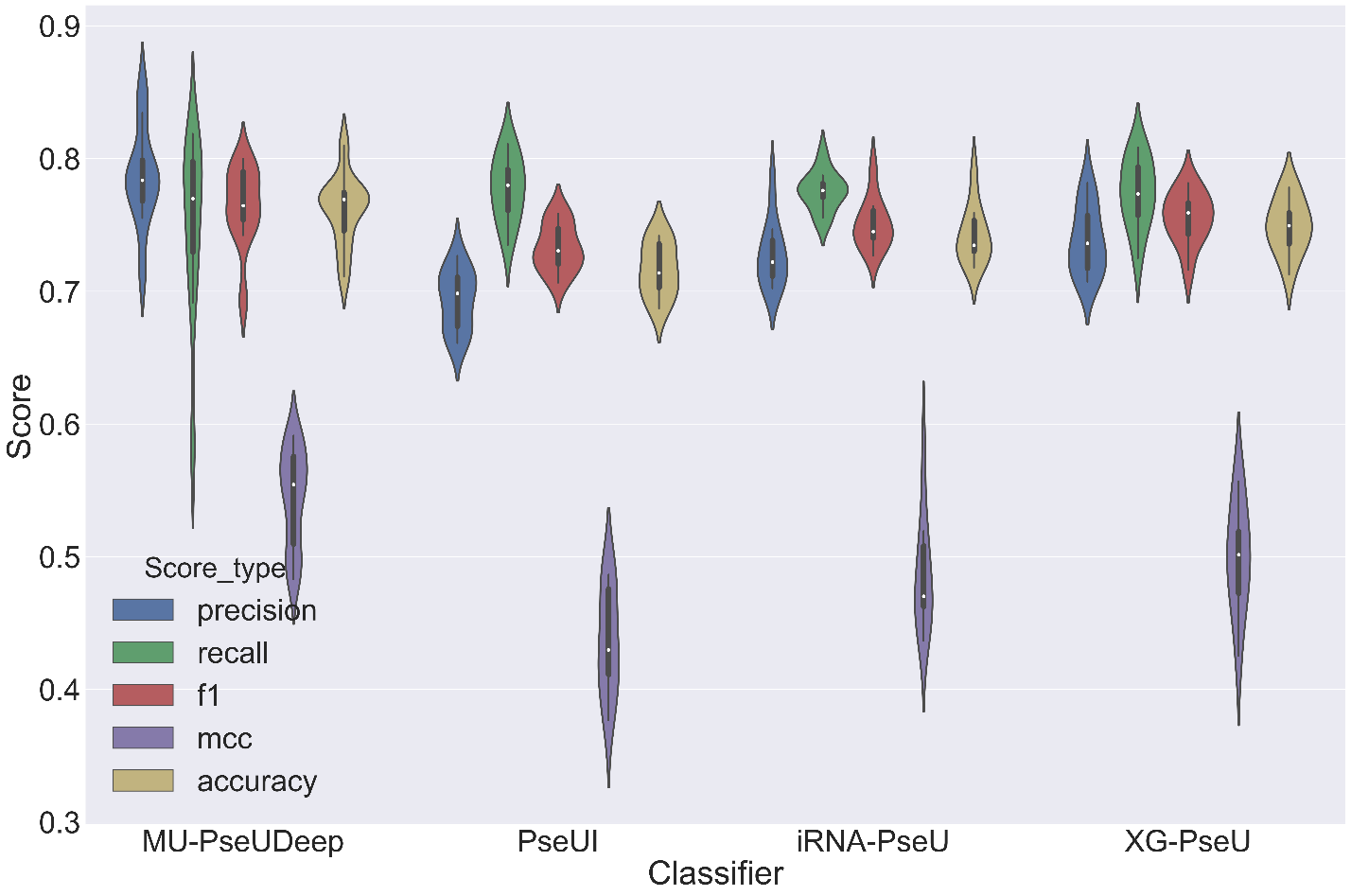
**

**(a)**


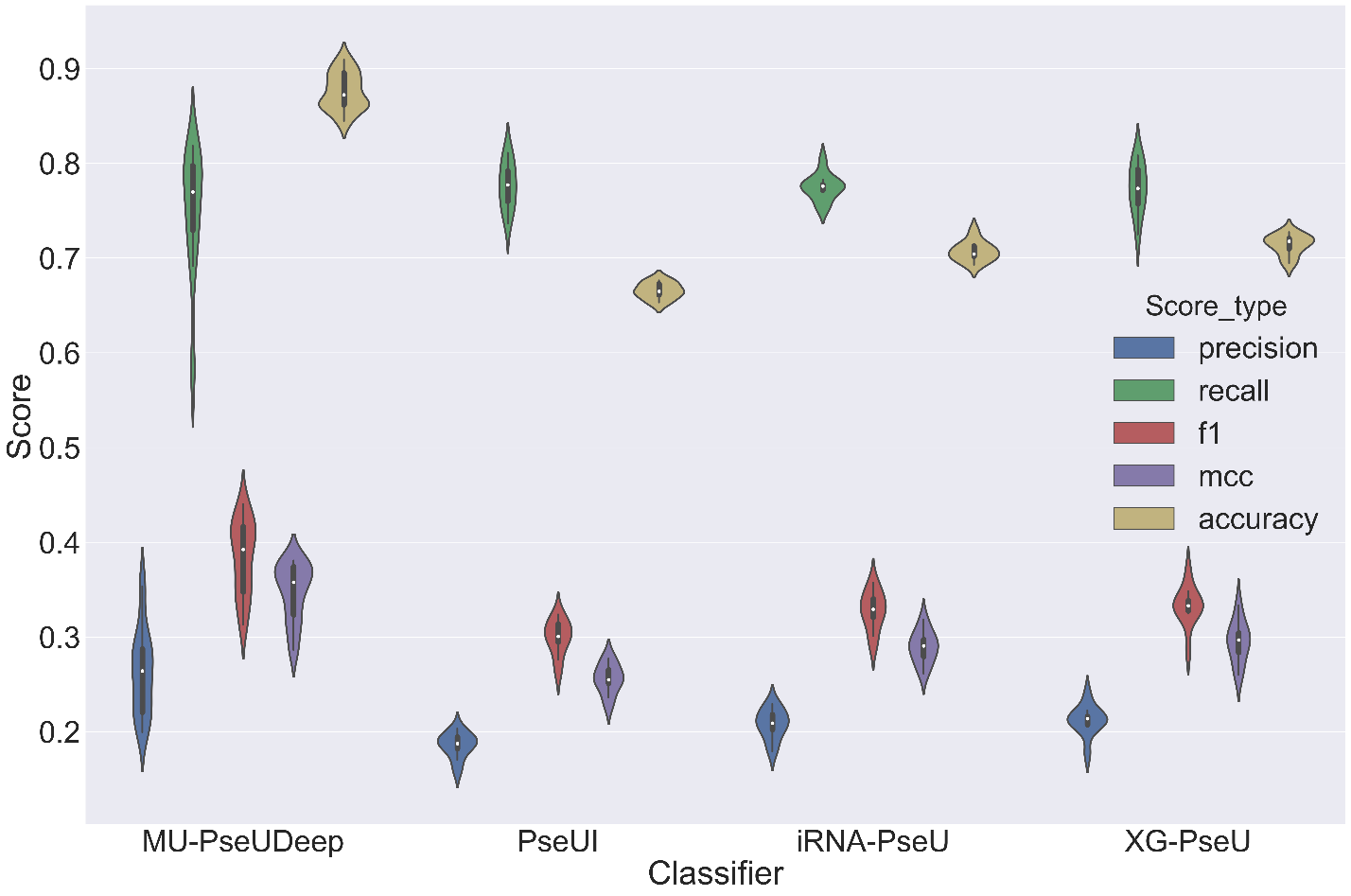


**(b)**

**Fig. S6. Performance comparison of deep learning method with other published methods on (a) balanced yeast data and (b) imbalanced yeast data**

**
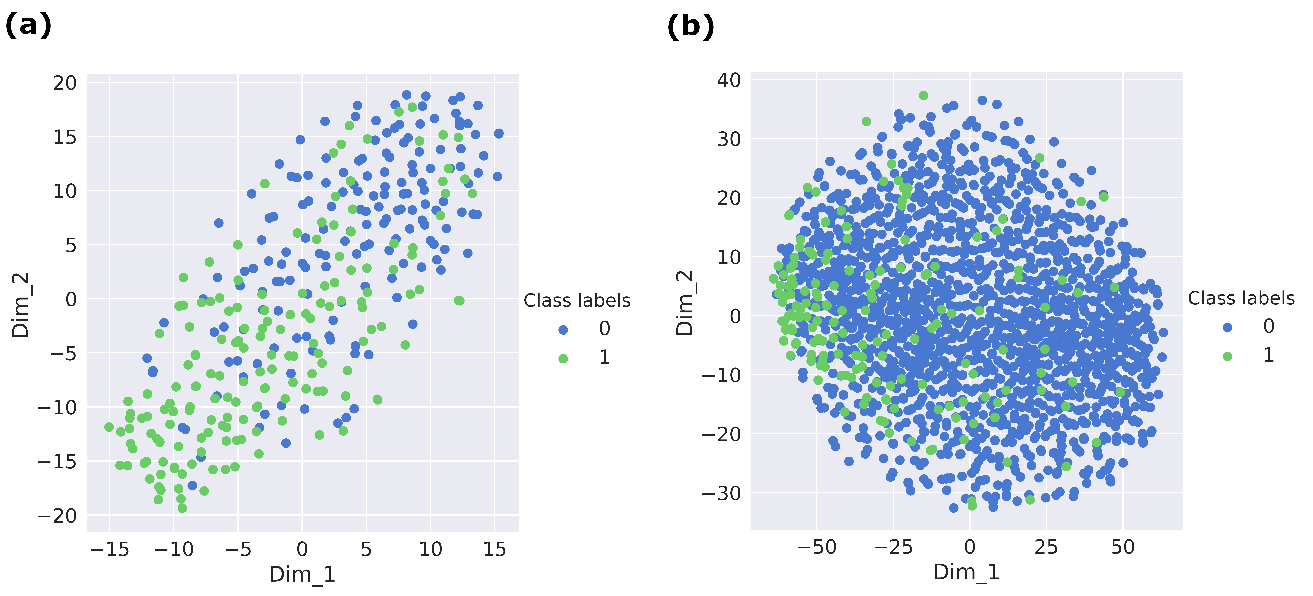
**

**Fig. S7. t-SNE plot yeast.** Visualization of the last feature map of separation between positive and negative class for

(a) balanced data and (b) imbalanced data.


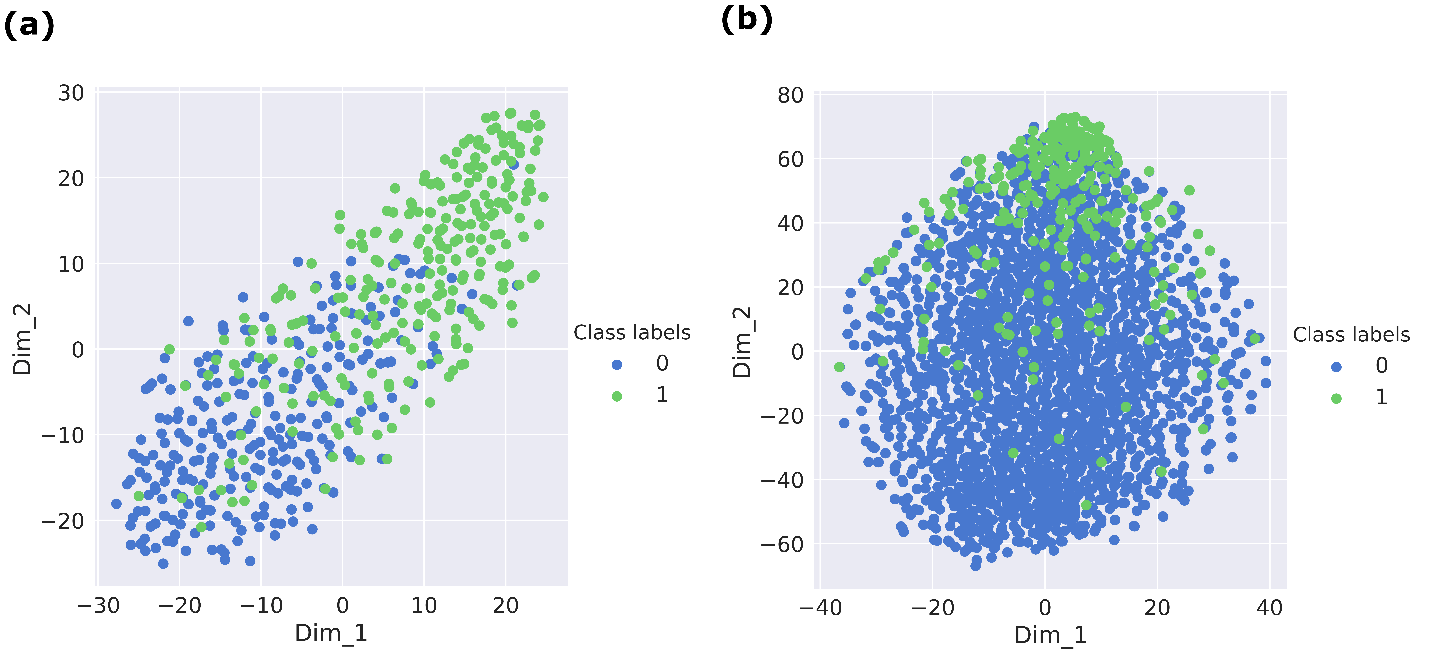


**Fig. S8. t-SNE plot mouse.** Visualization of the last feature map of separation between positive and negative class for

(a) balanced data and (b) imbalanced data.


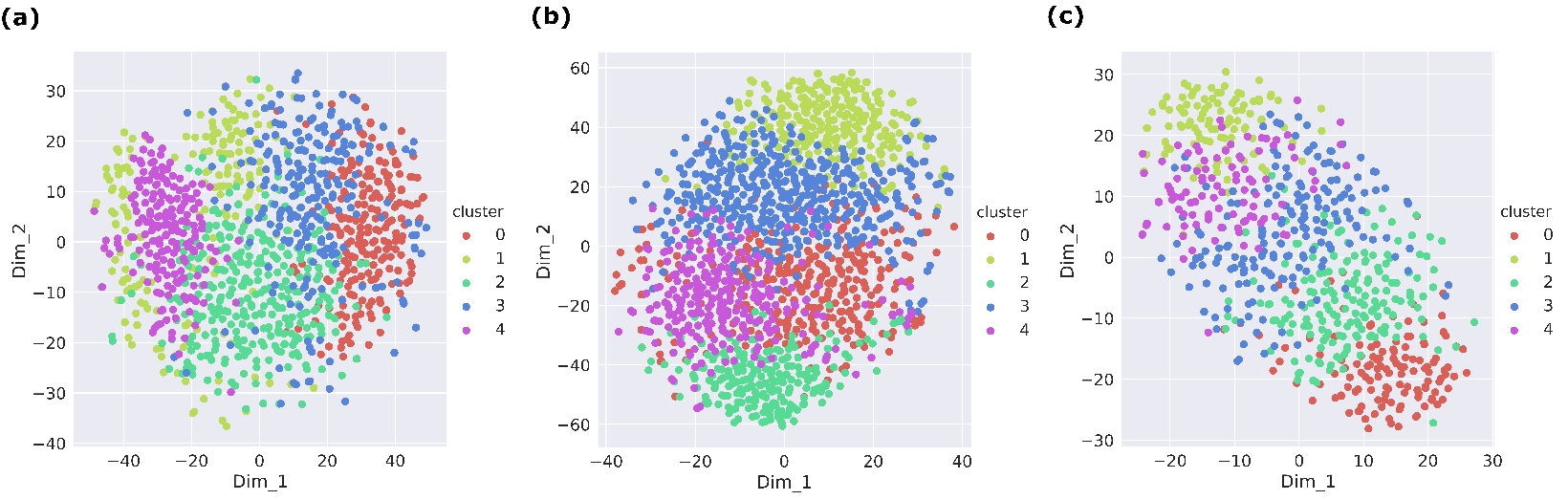
**Fig.**  **S9. t-SNE plot positive class clusters.** Feature map visualization of sequence clusters in positive class only for (a) human, (b) mouse, and (c) yeast data sets.

**
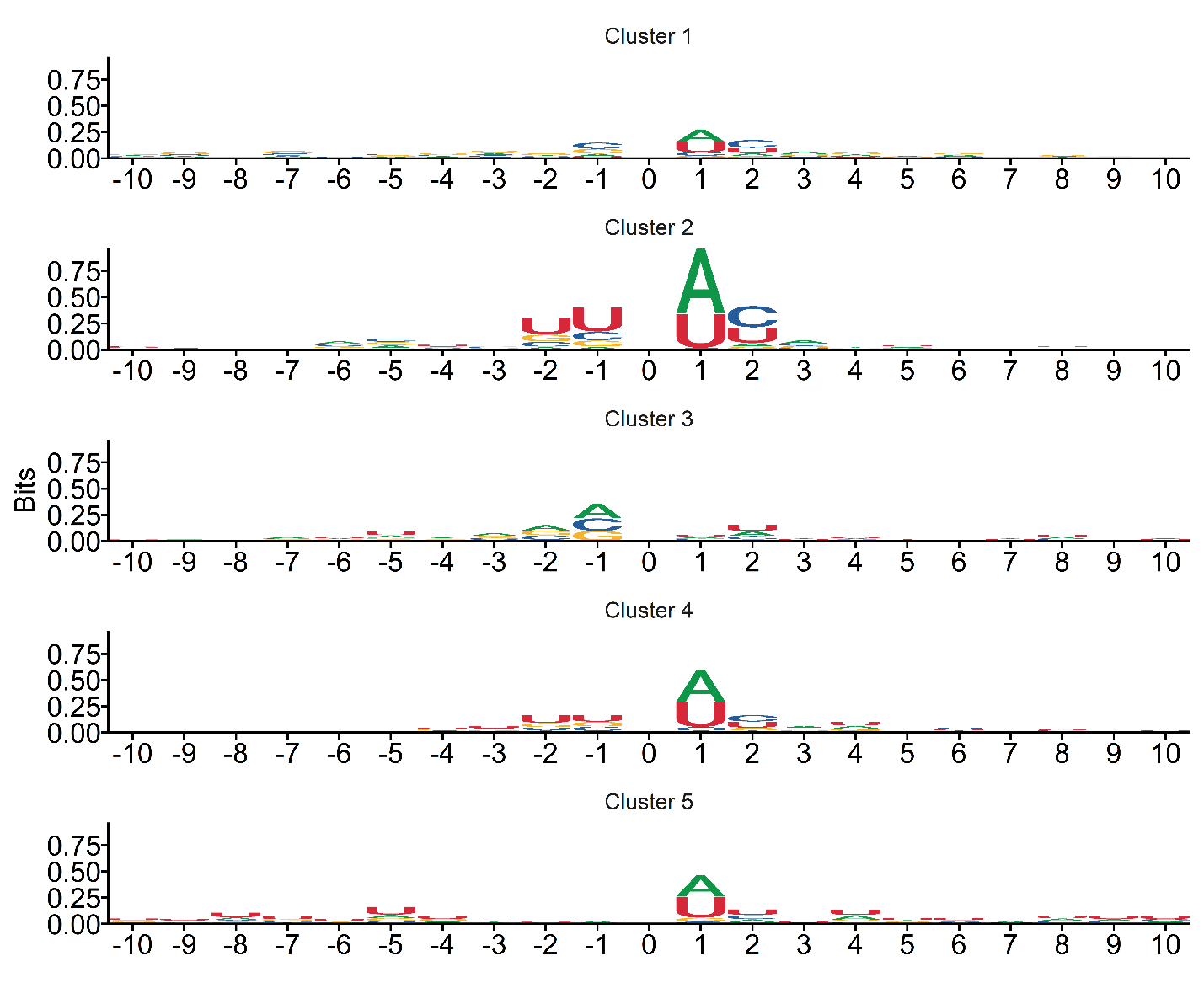
**

**Fig.**  **S10. Positive class sequence motifs.** Sequence motifs anterior and posterior to Ψ site in mouse.

**
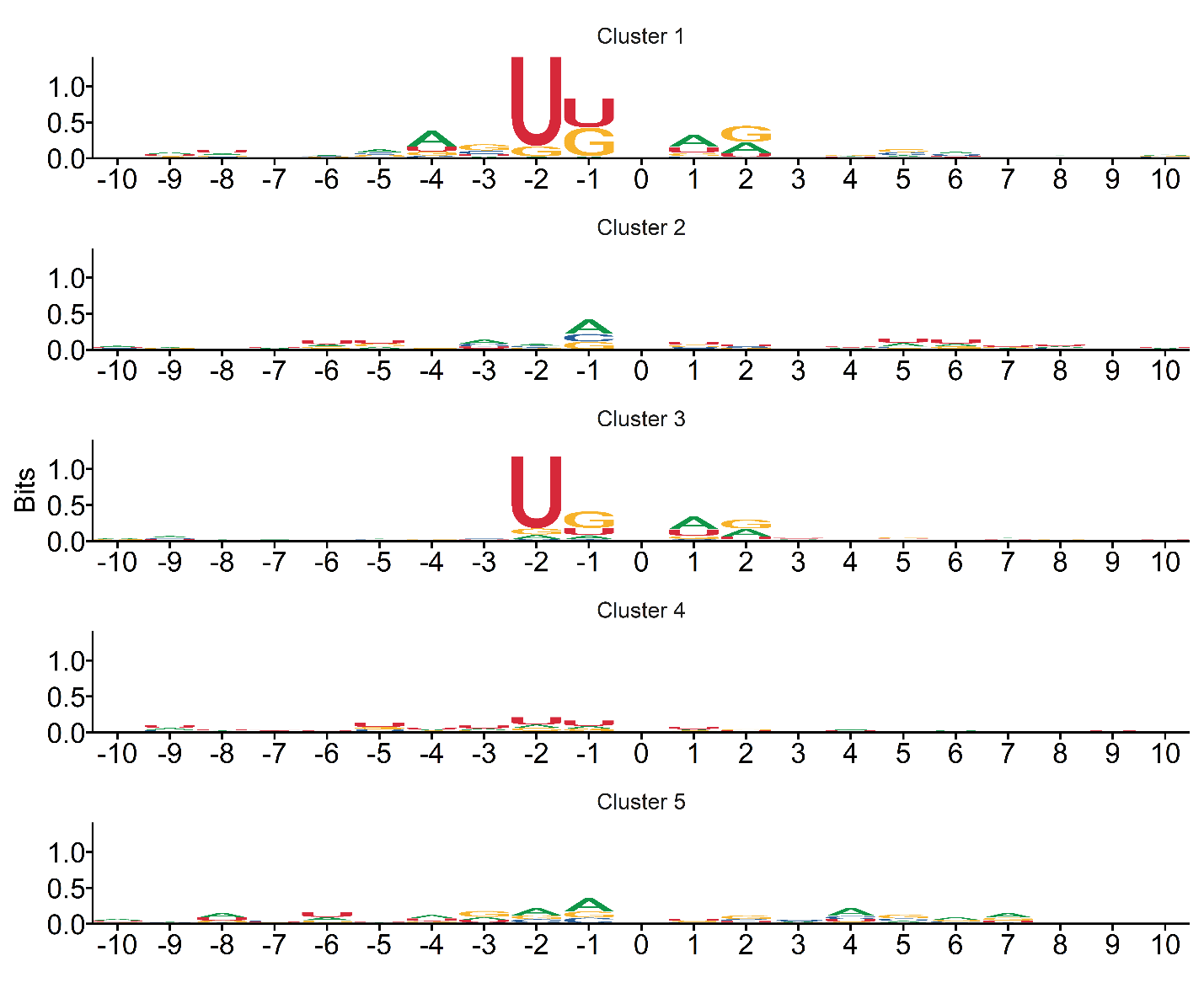
**

**Fig.**  **S11. Positive class sequence motifs.** Sequence motifs anterior and posterior to Ψ site in yeast.


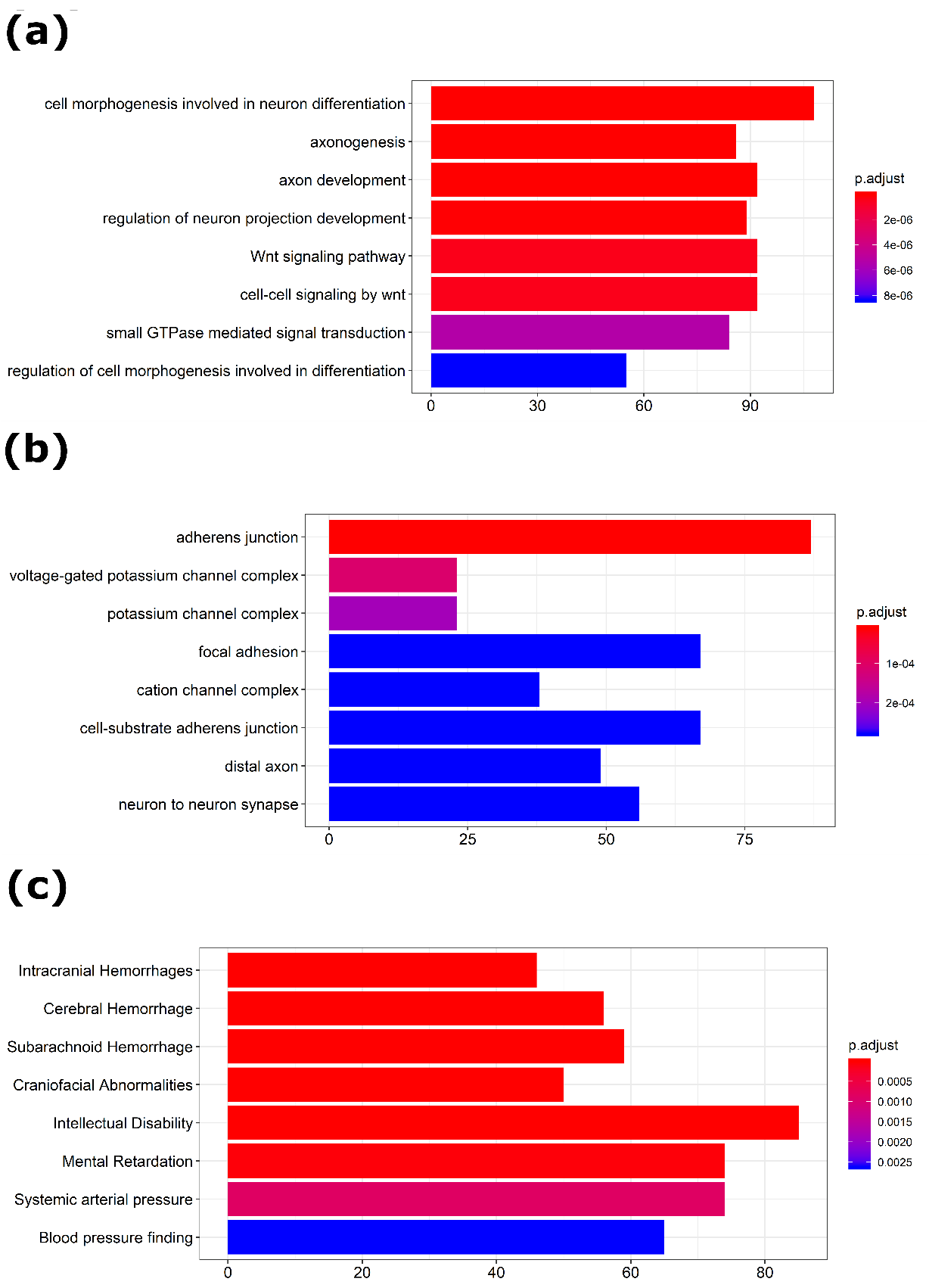


**Fig. S12. Enrichment analysis.** (a) Biological process enrichment, (b) cellular component enrichment,

and (c) disease gene network enrichment.

**
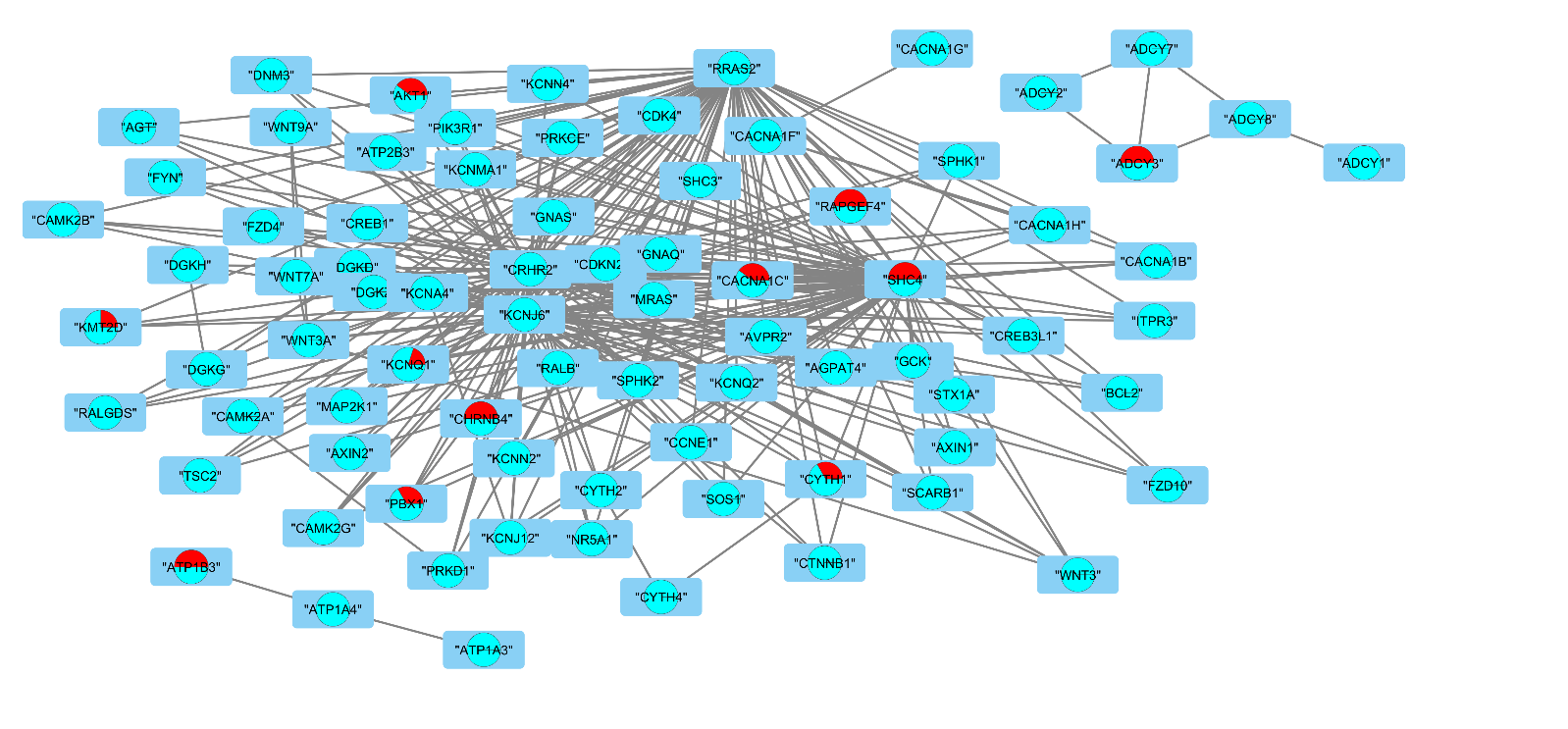
**

**(a)**

**
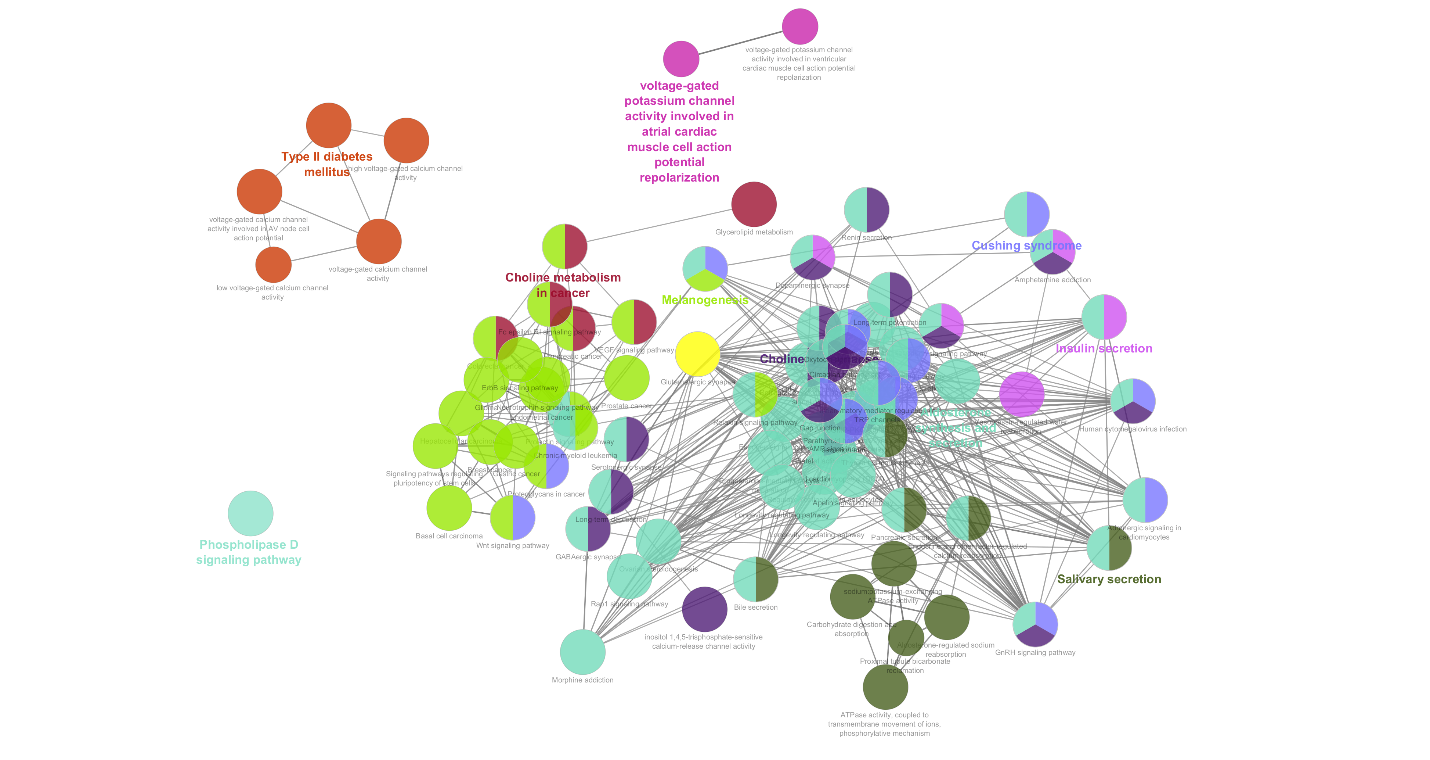
**

**(b)**

**Fig. S13. Gene functional network visualization. (a)** Genes with a GO semantic similarity score of > 0.9 were visualized in the network using Cytoscape. Each gene was visualized as a pie-chart with already known pseudouridine sites (represented by red) and pseudouridine sites predicted at > 0.99 threshold (represented by blue) **(b)** This network of genes was further visualized for the KEGG pathway and GO molecular function enrichment indicating some functional clusters of genes based on pathways and molecular functions.
